# New clues on the Atlantic eels spawning behavior and area: the Mid-Atlantic Ridge hypothesis

**DOI:** 10.1038/s41598-020-72916-5

**Published:** 2020-10-06

**Authors:** Yu-Lin K. Chang, Eric Feunteun, Yasumasa Miyazawa, Katsumi Tsukamoto

**Affiliations:** 1 Application Laboratory, Japan Agency for Marine-Earth Science and Technology, Yokohama, 236-0001 Japan; 2 BOREA (Museum National D’Histoire Naturelle, CNRS, Sorbonne Université, Université de Caen, IRD, Université des Antilles), MNHN, Station Marine de Dinard, CRESCO, 35800 Dinard, France; 3 Department of Aquatic Bioscience, The University of Tokyo, Tokyo, 113-8657 Japan

**Keywords:** Marine biology, Animal migration, Physical oceanography, Biogeography

## Abstract

The Sargasso Sea has long been considered as the only spawning area for Atlantic eels, despite the absence of direct observations. The present study raises a novel scenario, deviating from Schmidt’s dogma, begins with a review of historical and recent observations that were combined to build up a global theory on spawning ecology and migration behavior of Atlantic eels. From this, it is argued that a favorable spawning area could be located eastward of Sargasso Sea at the intersection between the Mid-Atlantic Ridge and the oceanic fronts. Ocean circulation models combined with 3D particle-tracking method confirmed that spawning at this specific area would result in larval distribution fitting the field observation. This study explores the hypothesis that leptocephali are able to swim and orientate to reach their specific growth areas. It proposes a novel framework about spawning ecology, based on orientation, navigation and meeting cues of silver eels to the spawning area. Together this framework may serve as a stepping-stone for solving the long-lasting mystery of eel reproduction which first came out 2,400 years ago and promotes the understanding of oceanic migration and reproduction of marine organisms.

## Introduction

Since Danish fishery biologist Johannes Schmidt’s renowned discovery in the twentieth century, the Sargasso Sea has been widely considered to be the Atlantic Ocean spawning area for the European eel *Anguilla anguilla* and American eel *Anguilla rostrata*^1^. After many research cruises collecting transparent leaf-like eel larvae known as leptocephali, Schmidt proposed a theory that eels in the Atlantic Ocean were born in the Sargasso Sea and then transported by currents to the Caribbean Sea and North American coastal waters in the case of American eels, and to the Mediterranean Sea and European coasts in the case of European eels. In recognition of this achievement, the United Kingdom’s Royal Society awarded Schmidt its Darwin Medal in 1930.

However, in 1959, Denys Tucker of the British Museum proposed a new hypothesis that ran contrary to Schmidt’s well-established theory. This hypothesis claimed that European eels were not distinct species, but were rather ecophenotypes of American eels and that differences in vertebral counts between these two types of eel could be caused by slight differences in water temperature during early development^2^. His hypothesis caused a major controversy among Europe’s scientific community that even included the Archbishop of Canterbury^3^. Finally, Atlantic anguillid population genetic data from protein electrophoretic analyses^3^ and recent DNA data^4–6^ definitively confirmed the existence of two distinct species. Thus, it is now widely accepted that two distinct eel species exist on each side of the Atlantic Ocean. Although Tucker’s hypothesis was itself denied, scientific discussion surrounding the hypothesis served to motivate considerable research into the natural history and biological and taxonomic status of Atlantic eels^4^.

**Figure 1:**
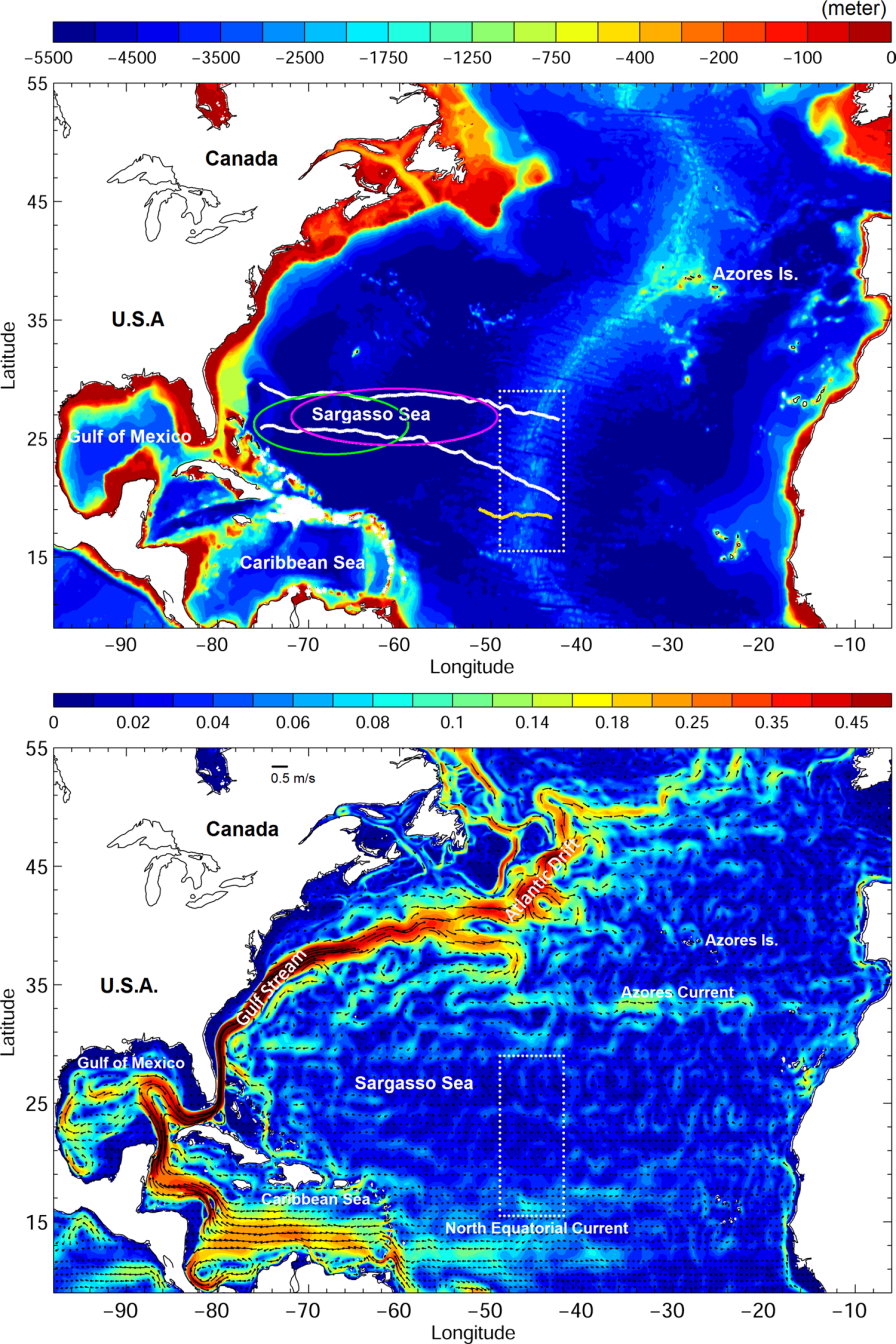
(top) North Atlantic Ocean bathymetry. White contours are averaged positions of 22 °C (north) and 24 °C (south) isotherms, and the yellow contour represents the mean position of a 36.9 psu salinity front. Green and magenta circles indicate areas where American and European eels have been observed, respectively^8^, and the white dotted box indicates the examined area for the numerical experiment. (bottom) 0–200 m averaged ocean circulation.

Schmidt’s conclusions of spawning area locations in the Sargasso Sea were later supported by further larva collection^7^ and reexamination of collection data^8, 9^. However, surveys for larvae and eggs had been confined to a relatively limited area, without extending to south and east of Schmidt’s hypothetic spawning area (magenta circles in Fig. 1 top)^9^. Despite a great effort in larval surveys in the Sargasso Sea over a century, eggs and spawning adults that could provide direct evidence of exact spawning locations within Sargasso Sea spawning areas have never been collected. In this context, spawning ecology of the European eel and American eel, including exact locations and lunar cycle timing, is not yet fully understood and relies on indirect evidence^10^. Hence, doubts about exact spawning areas in the Sargasso Sea have also been raised^11, 12^.

On the contrary, for the Japanese eel *A. japonica* in the Pacific Ocean, both eggs^13^ and spawning-condition adults^13–15^ have been collected in the spawning area along the West Mariana Ridge (seamount chain) in the western North Pacific. Those marine explorations lasted over six decades before eggs were collected^16^. The historical surveys were not limited in a specific region, the eggs were indeed caught hundreds and thousands of kilometers away from the early proposed region near Japan^17^. The discovery of the spawning area of Japanese eel resulted in the validation of many hypotheses intended to narrow-down the space and timing of spawning events through the analysis of historical leptocephalus catches and estimation of hatching dates using daily rings of otolith (ear stone). The results of several surveys since then indicated that spawning only occurred near the southern end of the West Mariana Ridge a few days before each new moon during April to August^17, 18^.

In addition, recent genetic research^19^ has suggested fragmented spawning areas for European eels, and otolith microchemistry^20^ evokes the putative existence of spawning sites other than the Sargasso Sea. This is illustrated by the high Mn concentrations at the center of otoliths of glass eels collected in western European estuaries suggesting that these eels spent their early life stages near areas influenced by the plumes of volcanic activity. However, no seamounts with volcanic activity exist in the hot-spot survey region in the Sargasso Sea. In this context, we see uncertainty about whether the Sargasso Sea from Schmidt’s Theory serves as the only spawning area for Atlantic eels.

The present study brings new insights on the location of Atlantic eels spawning areas by proposing arguments based on historical observations to elaborate a global theory on spawning ecology of Atlantic eels, with new clues on orientation and meeting behaviors of silver eels on their spawning migration. We also test an alternative Atlantic eel spawning area using numerical simulation that includes swimming and orientation behaviors of leptocephali. The numerical simulations showed a similar larval distribution to observation, whether the departure of virtual larvae (v-larvae) are included within or outside the historical Sargasso Sea spawning areas. We are aware that our research may deeply challenge a well-established theory; however, we intend to provide a strong scientific contribution to marine biology, oceanography, and fisheries science by facilitating a broad intensive discussion related to spawning of Atlantic eel, which would enhance understanding of a primary mechanism of fish migration and reproduction in the ocean.

## Arguments and discussion

To enable successful spawning of Atlantic eels in remote offshore areas of the ocean, three conditions need to be met. This requires, first, appropriate navigation abilities and cues leading to the remote spawning area; second, a meeting point; and third, an adequate timing.

### Orientation and navigation cues towards the spawning area

Eels are thought to imprint a magnetic map on their first transoceanic migration from the spawning areas to the coasts^21^. Moreover, silver eels are known to be sensitive to magnetic cues^22^ that are likely involved in navigation towards the Sargasso sea with a very high spatial accuracy^23^. Under this hypothesis, silver eels are expected to choose the fastest or shortest route to join the Sargasso Sea. Indeed, recent studies showed that European silver eel swam south-westward^24, 25^ while American silver eels swam south-eastward^26^. Surprisingly, none of the tagged eels reached the spawning areas within the Sargasso Sea^26^. One single American eel reached the north west boundaries of the North Atlantic Convergence zone at > 2,000 km from the center of the Sargasso Sea^26^, while a few European eels where detected at the North East of the Azores at c.a. 3,000 km from the Sargasso Sea^24, 25^. This was interpreted as a consequence of the tagging rather than a biological fact.

Interestingly, all European eels, whatever their release points (Baltic Sea, Ireland, the Bay of Biscay, Mediterranean) converged towards the Azores, which is not the shortest way back to the Sargasso Sea^24^. So what could be the advantage for silver eels for choosing a longer route? The most parsimonious hypothesis is that the Azores serve as a meeting point located along the Mid-Atlantic Ridge. Once they reach this point they turn southwest, following the Mid-Atlantic Ridge. This could be made possible by the striking vertical diel migration behavior that takes eels from epipelagic layers (150–300 m) during the night to mesopelagic and bathypelagic depths during the daytime (down to 1,200 m)^24–26^. This behavior could enable silver eels to detect and follow the Mid Atlantic Ridge and associated seamounts that culminate at 2,000 m to 3,500 m above the seafloor that lies at > 4,000 m depths. Moreover, it is likely that eels detect chemical variations of the seawater using their high olfactory abilities enabling them to detect specific odors or plumes from subducted or convected deep layer waters^27, 28^. Indeed, the volcanic activity and deep currents disturbed by the sea level rise around the ridge likely modify the chemical composition and related odor of the water thus providing signposts^28^.

Following this north south Y axis, silver eels may finally reach favorable thermic conditions of 22 to 24 °C to spawn, which are located between two parallel east–west thermal fronts that occur in the Sargasso Sea at about 24°N and 28°N (X-axis)^8, 29^. Worth mentioning, small leptocephali of both Atlantic eel species have been collected over a wide longitudinal range (75–50°W) between these two fronts^8^. Although the collected area of American and European eel larvae partly overlapped in Sargasso Sea, the southern-most collection of European eel larvae was about 100–200 km north compared to American eel larvae^8^, apart from thermal fronts that were suggested earlier as an X-axis, European eels may follow a different hint. One of the major water masses in the Sargasso Sea is the North Atlantic Subtropical Mode Water, which has unique vertical temperature distribution, in which the temperature is nearly uniform in the Mode Water layer, especially in winter and early spring^30, 31^. Its southern boundary is around 22–26°N; therefore, the mode water’s boundary could also potentially serve as a destination hint (X-axis) for European eels.

### Meeting point to mitigate lack of migration timing

Once eels have reached favorable habitat conditions to spawn, they have to find their mates to breed. Random mating in the huge Sargasso Sea (c.a. 3 million km^2^) is highly unlikely. Indeed, male and female silver eels do not have a synchronized migration. Males start their migration from August to September, whereas females migrate between November and December^24^. Telemetry data demonstrated that migrating silver eels disperse after they are released^24^. Migration speed is highly variable according to size^24, 32^, because males that are approximately 45 cm long on average have much lower swimming speeds than female eels, which have bodies up to twice the size as males. This suggests that, unlike tuna or mackerel, eels do not form schools, and even if they start their spawning migration in a school from continental rivers, they eventually scatter and arrive in the Sargasso Sea one by one. These arguments strongly suggest that synchronized migration and schooling do not likely occur, meaning that successful mating and spawning depends on the existence of clear physical, chemical, geological, or biological signals that eels can use to locate a meeting point in the ocean. However, such east–west and north–south hints (X and Y axis) or any kind of gradient do not exist in the large Sargasso Sea.

Egg distributions of Japanese eel within the spawning area indicated that spawning occurred just south of the crossing point where north–south seamount chain and east–west salinity front between two water masses with different salinities—caused by evaporation in the north and tropical rainfall in the south^13, 16^. It has been speculated that eels can locate the spawning site using a combination of the seamount chain (Y-axis) and salinity front (X-axis) as a signpost for forming spawning aggregations in the ocean.

To ensure successful external fertilization of eggs, eels must meet their mates in the ocean, meaning that time and space must precisely coincide for successful mating. If the same strategy can be adapted to Atlantic eels, waters near the Mid-Atlantic Ridge could be chosen as a spawning site because of unusual topographical features, geomagnetic anomalies^33^ or variation of chemical compositions that could serve as an olfactory cue for eels. Indeed, active hydrothermal vents have been observed along the Mid-Atlantic Ridge across the entire Atlantic^34^, and the release of chemical elements from hydrothermal vents may serve as a cue for locating a spawning site. This kind of signpost remains very large, and therefore it is likely that pheromones might be released by silver eels to favor the final meeting of the partners.

### Simulating departure from the Mid Atlantic Ridge and from the Sargasso Sea

Using the same principle as Japanese eel, volcanically active parts of the Mid-Atlantic Ridge could be one of the spawning sites for Atlantic eels due to unusual topographical features, geomagnetic anomalies, or differing water chemical composition^21^. The 22 °C and 24 °C thermal fronts between which Atlantic eel larvae have been frequently observed^8^ are used to extend farther east, interacting with the Mid-Atlantic Ridge at around 27 and 20°N, respectively. To the south of these thermal fronts exists a discernible salinity front around the northern limit of the North Equatorial Current (NEC) in the Atlantic at 15–18°N. Thus, we modeled the transport of virtual leptocephali larvae from the area chosen to be 15–29°N and 43–48°W which included intersections of the Mid-Atlantic Ridge by one salinity front and two thermal fronts (Fig. 1 top).

**Figure 2:**
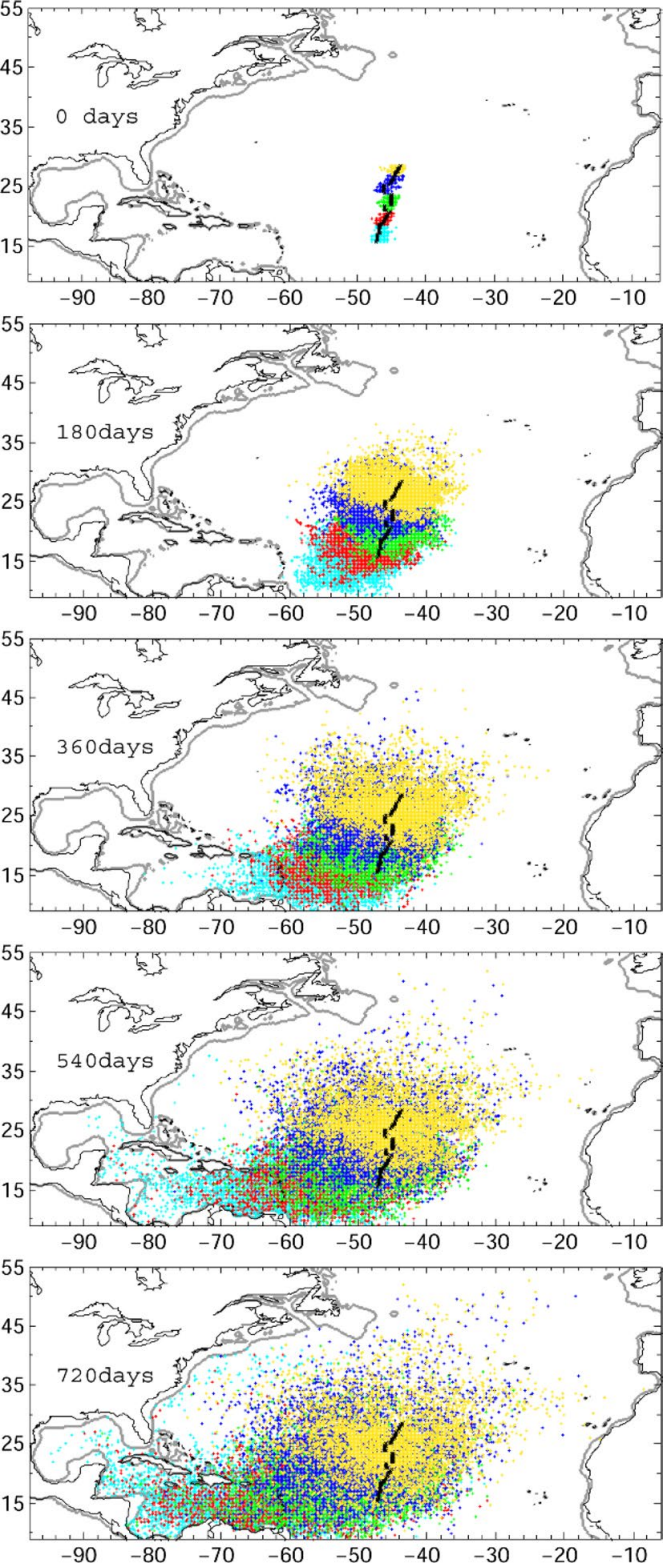
Distribution of passive swimming v-larvae departing from near the Mid-Atlantic Ridge. Colors correspond to release areas (north of the 22 °C isotherm (yellow), between the 22 and 23 °C isotherm (blue), between the 23 and 24 °C isotherm (green), south of the 24 °C isotherm (red), and the north of NEC with a salinity front at around 18–19°N (cyan)) as indicated in the top panel. The simulation period was 1993–2000 and included both positive (1993–1994, 1999–2000) and negative (1995–1996, 1997–1998) North Atlantic Oscillation events, and the results are based on an eight-year composite.

We then released v-larvae near the Mid-Atlantic Ridge from 15 to 29°N. We classified v-larvae by their initial positions as north of the 22 °C isotherm (yellow), between the 22 and 23 °C isotherm (blue), between the 23 and 24 °C isotherm (green), south of the 24 °C isotherm (red), and the north of NEC with a salinity front at around 18–19°N (cyan) (Figs. 1, 2). Passive swimming v-larvae were widely dispersed to the west and east of the release area after 720 days of migration (Fig. 2). V-larvae departing from north of 24°N (yellow and blue dots) finally arrived at the Azores front and North Atlantic drift, with easternmost positions near 15°W, showing similar distribution to observed European eel larvae. In contrast, v-larvae departing from south of 24°N (green, red, and cyan dots) could make it to the Caribbean Sea and the Gulf of Mexico, and some v-larvae entrained in the Loop Current and Gulf Stream, arriving at the east coast of North America, that is similar to the observed American eel larvae distribution. The percentage of v-larvae reaching 25°W after 720 days decreased from north to south: 0.71% in the northernmost area (yellow in Fig. 2), 0.13% (blue), and 0% (green, red, and cyan). Arrival at the Caribbean Sea and Gulf of Mexico increased from north to south: 0.13% (yellow in Fig. 2), 0.77% (blue), 4.64% (green), 19.3% (red), and 38.9% in cyan.

**Figure 3:**
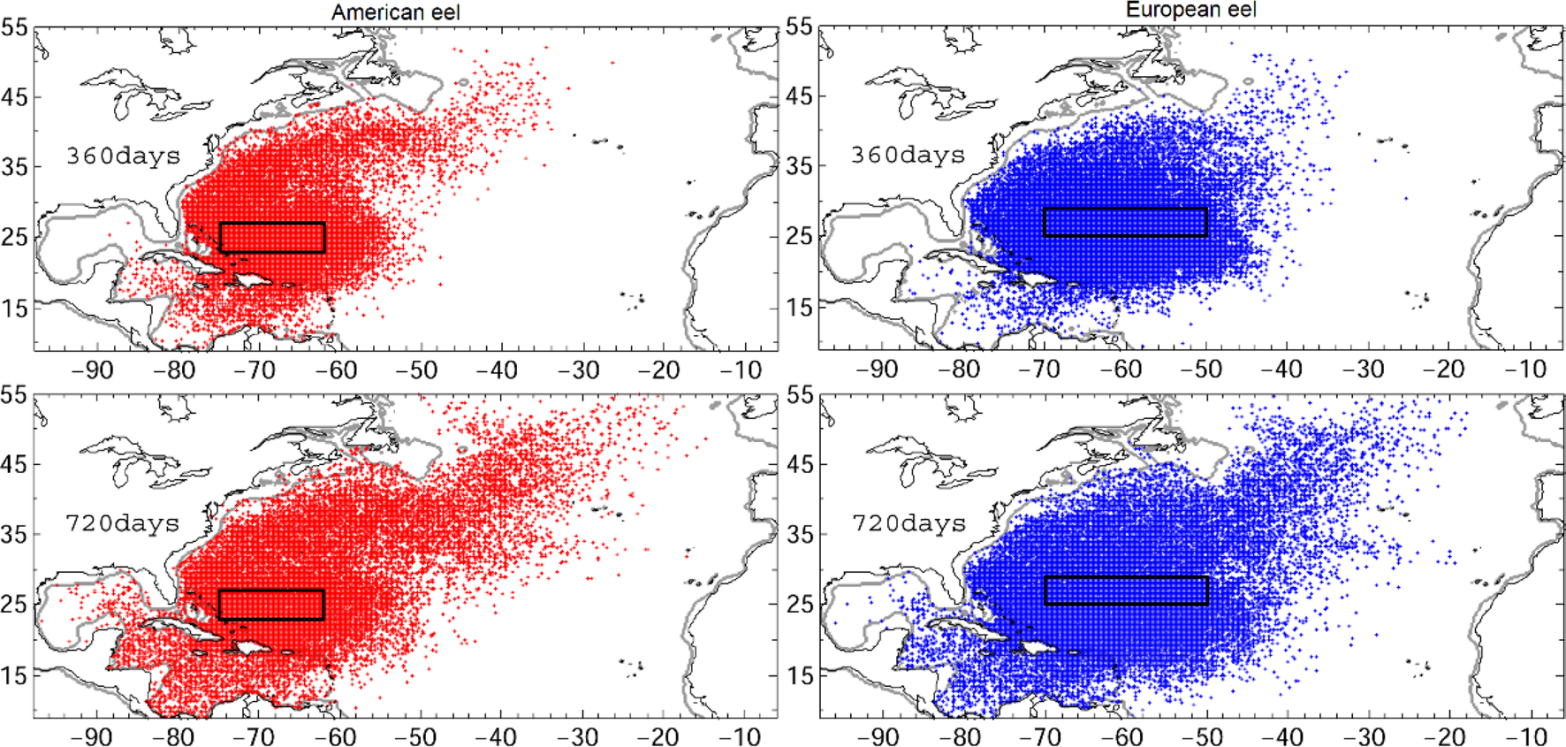
Distribution of v-larvae released in the Sargasso Sea for American (left) and European eels (right).

By comparison, v-larvae released in the Sargasso Sea were widely distributed throughout the northwestern Atlantic Ocean, including the Caribbean Sea and Gulf of Mexico (Fig. 3). A total of 0.14% of the v-larvae released from the suggested European eel spawning area in the Sargasso Sea reached 25°W after 720 days (Fig. 3 right), whereas 0.27% of those released in the American eel spawning area reached 25°W (Fig. 3 left). Arrival at the Caribbean Sea and Gulf of Mexico was 6.56% and 11.9% of those released from European and American eel spawning areas, respectively.

Although the distribution patterns were similar to v-larvae departing from the newly proposed spawning area (Figs. 3, 5), differences were detected. A significant fraction of v-larvae representing European eels released in the Sargasso Sea dispersed to the Caribbean Sea and Gulf of Mexico (Fig. 3, right). As the v-larvae departing from the northern proposed area did not enter these areas (Fig. 2), and only American eel larvae but not European eel larvae have been collected in the Caribbean Sea and Gulf of Mexico. In addition, some of v-larvae representing American eel departing from Sargasso Sea were transported far northeast by Gulf Stream and North Atlantic Drift to east of 40°W, where American eel larvae were not observed^8^. In contrast, v-larvae departing from southern proposed area showed closer distribution to observations of American eel leptocephali, while v-larvae departing from central to northern sub areas of the Mid-Atlantic Ridge presented similar distributions to observations of European eel leptocephali. Therefore, it could be suggested that both European and American eels may indeed spawn in the newly proposed area near the Mid-Atlantic Ridge.

**Figure 4:**
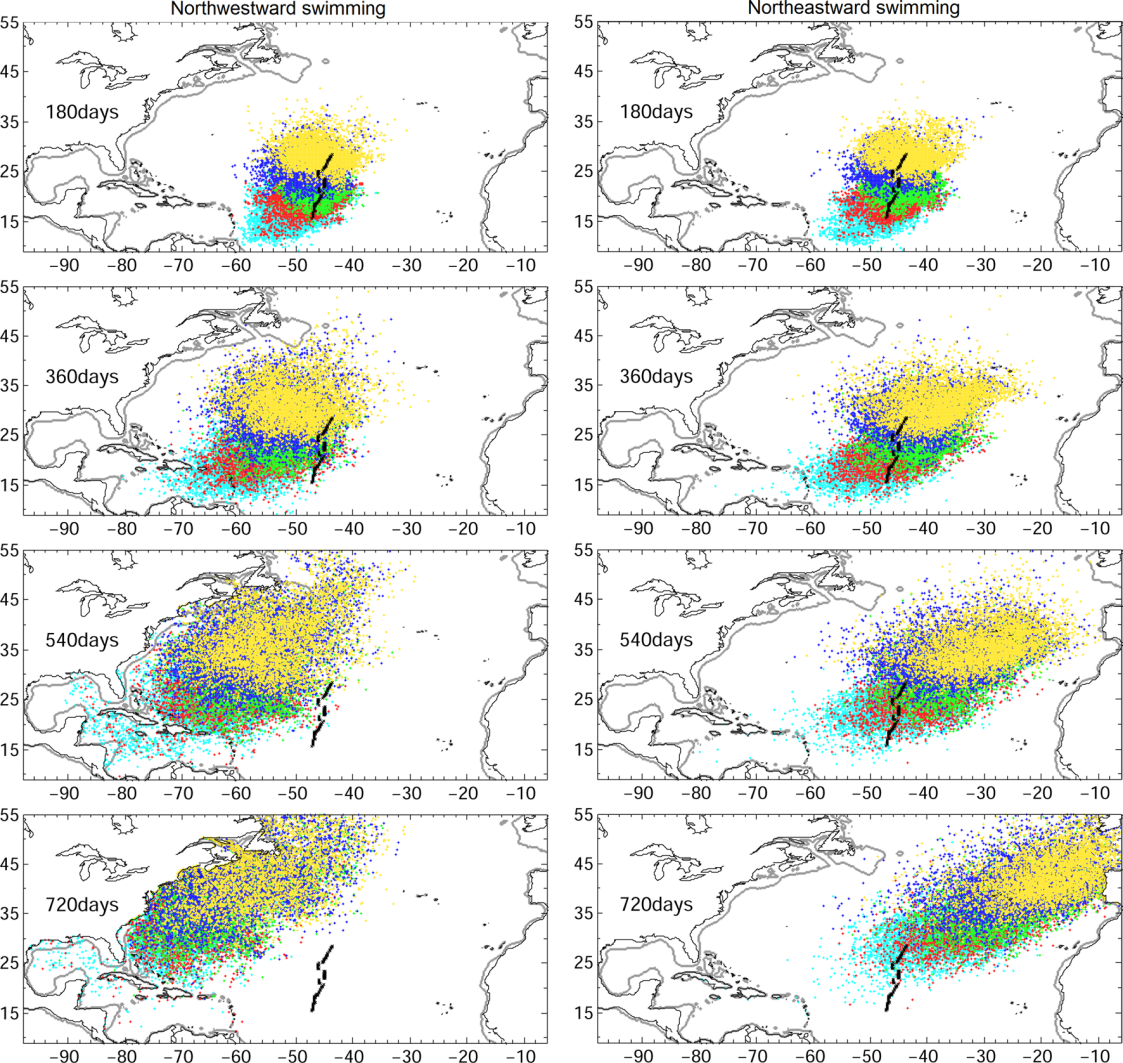
Same as Fig. 2, but for northwestward swimming (left), and northeastward swimming (right) at swimming speed of 1 BL/s.

Interestingly, distributions of v-larvae departing from the American eel spawning area or from the European eel are very similar (Fig. 3) suggesting that swimming and orientations are likely. If v-larvae could swim at 1 body length per second (BL/s) northeastward, arrival rate at 25°W would increase substantially, especially for those departing from the northern area (Fig. 4, left). On the other hand, v-larvae swimming at the same speed of 1BL/s but heading northwestward would not reach 25°W (Fig. 4, right), instead, distribution of v-larvae would be concentrated at northwestern Atlantic Ocean. The simulations with swimming ability indeed also revealed similar distribution as observations. V-larvae departure from northern area would swim towards eastern north Atlantic (yellow and blue, Fig. 4 left), whereas those departing from southern area (cyan and red, Fig. 4 right) would move towards western north Atlantic and some of them may bypass Caribbean Sea and Gulf of Mexico.

### Estimating spawning location of small leptocephali caught in historical surveys

Historical surveys have spent great effort to search the eggs and spawning adult eels in the past century. However, the larval surveys to date have not explicitly considered the possibility of alternative spawning areas or an extension eastward. This introduced an evident gap, both geographically and temporally, in larval surveys. Indeed, our numerical simulation showed that a different departure point (spawning area), located above the Mid-Atlantic Ridge, resulted in a distribution of leptocephali larvae similar to historical observations in the Atlantic Ocean. Hence, these results strongly suggest that oceanographic surveys should be organized outside the Sargasso Sea, in the vicinity of the Mid Atlantic Ridge.

**Figure 5:**
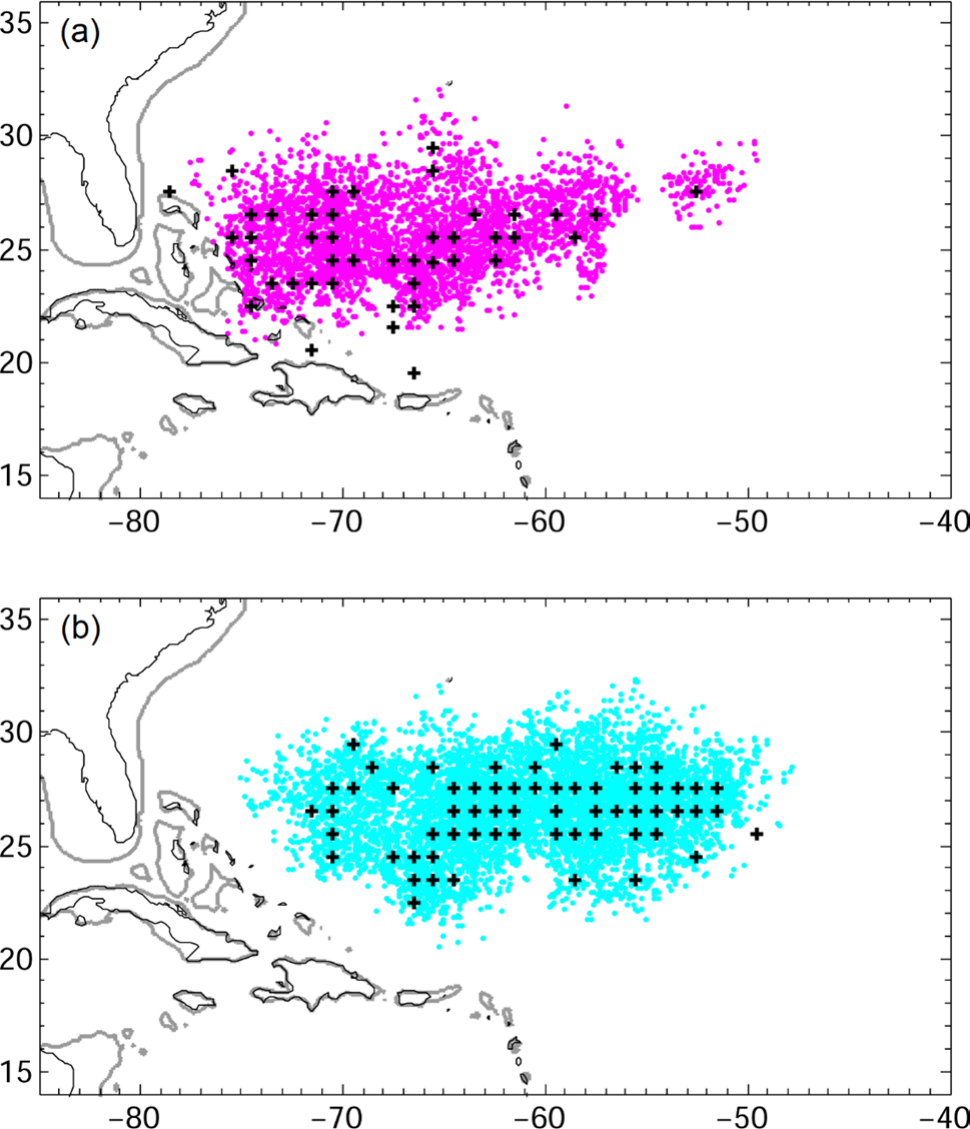
Distributions of passive backward tracking v-larvae 30 days prior to collection for (**a**) American eels, and (**b**) European eels. Black crosses showed the released locations that followed the positions where eel larvae were collected^8^.

We applied passive backward particle tracking to trace the origin of those observed Atlantic eel larvae. We released v-larvae in the Sargasso Sea where ≤ 10.9 mm Atlantic eel larvae have been collected^8^. The distribution of potential v-larvae origins 30 days prior to on-site collections was not far from where eel larvae have been collected (Fig. 5), as ocean currents were rather weak and lacked a unified direction. The results suggest a few possibilities, such as eggs may occur nearby the area where eel larvae were collected although they have not been collected; eel larvae indeed were observed in rather a wide region, suggesting eel larvae (or eggs) may also occur in areas located outside the hot-spot survey zone of the Sargasso Sea. Learning from the experience of Japanese eel surveys would allow exploring the hypothesis of alternative spawning locations.

### Disagreement from microchemistry aspect

**Figure 6:**
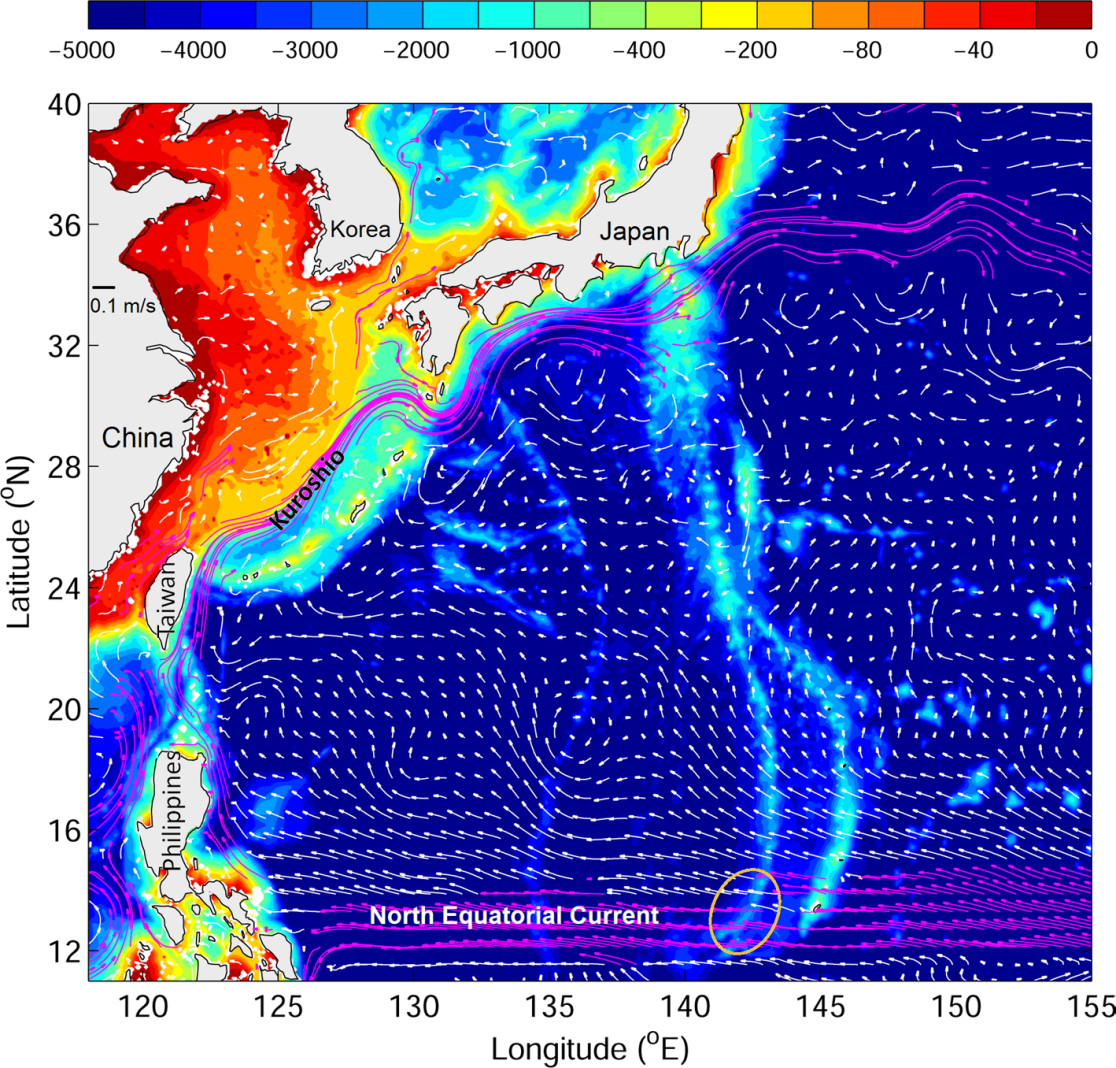
Bathymetry (shading) and mean ocean circulation (vectors) in the western Pacific. Fast and slow currents with criteria of 0.15 m/s are indicated by magenta and white vectors, respectively. The yellow circle marks the spawning area of Japanese eels. See the analogy of ocean current systems in both the Atlantic (Fig. 1) and Pacific (Fig. 6), i.e., the relationship between possible eel spawning locations and currents in the western subtropical gyre such as the North Equatorial Current and western boundary currents (Gulf Stream or Kuroshio).

Ocean currents in the Sargasso Sea are generally weak, with average speeds less than 5 cm/s in the top 200 m (Fig. 1 bottom). Eddy activity is inactive in the Sargasso Sea and eddy nonlinearity is relatively low compared to those formed near the Gulf Stream or Azores Current^35^, indicating less trapping and transporting by westward propagating eddies for marine organisms. Additionally, Japanese eels are spawned in the faster (10–20 cm/s) NEC in the Pacific (Fig. 6), thus, it can be said that the Sargasso Sea, which is the presumed Atlantic eel spawning area, is relatively quiet and has less transporting ability because of a subtropical gyre convergence zone. This convergence zone is unfavorable for the transport of eel larvae to continental rivers.

Interestingly, concentrations of Mn, a trace element signature, in the central part of otoliths of glass eels caught in western European estuaries were significantly greater than those of leptocephali collected in the Sargasso Sea^20^. Mn is a geochemical fingerprint of volcanic activity mainly found along the Mid-Atlantic Ridge^34^.The numerical experiment had shown that the buoyant hydrothermal plume could transport dissolved elements vertically by 1000–1500 m, and could also spread thousands of kilometers by horizontal advection. This suggests that glass eels caught in European estuaries spent their early life in the plume of a volcanic activity zone whereas leptocephali born in the Sargasso Sea may not successfully migrate to Europe due to being trapped in the convergence zone. This supports the existence of multiple spawning areas or batches suggested by Baltazar-Soares^36^, without affecting the well-established panmixia^36–39^ given that larvae seeded from any location are dispersed along with large recovering areas (Figs. 2, 4).

### General discussion

The present study explores the existence of another spawning area near the Mid-Atlantic Ridge at the east of the Sargasso Sea, that has been assumed to be the sole spawning area for almost 100 years since Schmidt’s research. This scenario relies on the combination of ecological and environmental inferences, comparative biology, the need of biotracers to pilot the catadromous fish and modelling.

The distribution of v-larvae departing from the newly proposed spawning area near the Mid-Atlantic Ridge showed possibilities of successful migration of both species to their respective geographic continental distributions. The v-larvae released in the northern region of the newly proposed spawning area showed distributions similar to those of collected European eel larvae, whereas those that departed from the southern region, within the salinity front, had distributions closer to the those of American eel larvae^8^. These fits between our model and observations of larval distribution are even stronger when assigning orientation and swimming skills to v-larvae. We therefore assume that swimming and orientation behaviors likely occur supporting previous findings and hypothesis^21, 40^.

Salinity fronts have been suggested to be related to Japanese eel spawning^41^ in the Pacific Ocean. Indeed, approximately 600 eggs have been collected over five research cruises at the intersection of the salinity front and the West Mariana Ridge. Similarly, a salinity front has also been observed in the Atlantic Ocean between the Sargasso Sea and NEC at around 15–18°N. In the new spawning area tested in this study, a strong salinity front with a rapid increased from 36.3 PSU at 15°N to 36.9 PSU at 19°N down to depths around 200 m was observed, and the front extended below 200 m south of 18°N. The salinity front could potentially provide a landmark for silver eels during breeding migration. In this study, v-larvae released near the salinity front showed quick dispersion westward, entering the Caribbean Sea and the Gulf of Mexico with some going to the Gulf Stream. This pattern is similar to that of Japanese eels in the Pacific Ocean that established their migration loop in the southwest corner of the subtropical gyre using the NEC and the Kuroshio^42^ (Fig. 6).

For the migration of adult eels, routes proposed by Righton et al.^24^ from a pop-up tag study showed that silver European eels seemed to converge toward the Azores regardless of origin (Baltic, North Sea, Celtic Sea, Bay of Biscay, Mediterranean). This does not fit with the Sargasso Sea hypothesis as the most direct routes from northern Europe and the Mediterranean to the Sargasso Sea do not encompass the Azores. Our hypothesis is that the Azores acts as a landmark for silver eels swimming southwest.

On their spawning migration, silver eels need to find the most efficient way to reach the spawning area using the safest and less energy costly route. It could be suggested that silver eels simply backtrack the migration route they used as leptocephali. This would imply that eels imprint their larval route, and that silver eels would have to swim against the strongest currents of the North Atlantic Ocean as the Gulf Stream, the Azores Currents and the North Atlantic drift (ie Miller and Tsukamoto^43^). This strategy would probably cost too much energy. Alternatively, by converging towards the Azores, as suggested by Righton et al.^24^, Silver eels avoid the strongest marine currents thus saving energy expenditures, which is a more likely evolutionary scenario. However, this would involve the existence of a genetically imprinted geomagnetic map that would enable eels to navigate towards the Azores whatever their departure point. Although possible, this assumption remains speculative as to date, science has not addressed how DNA encodes for such a behavior.

Because of their diel vertical migration ranging from ~ 800 m during the day to 300 m at night^24, 25, 44^, these eels could detect the topography and specific odors of the ridge they follow until they reach to favorable thermal fronts. Strong magnetic abnormalities occur along the Mid-Atlantic Ridge from the Azores to the junction with the Kane fracture zone (23.5 N; 46.4 W) and then make a bend westward along the Krane fracture^33^. For the American eel, an individual released from the Gulf of St. Lawrence near the northernmost distributional range of American eel leptocephali showed a long-distance migration to the northern Sargasso Sea^26^. We need to further observe the route in the southern Sargasso Sea. Additionally, the release of silver eels with pop-up tags from the Caribbean Sea near the southernmost distributional range and nearest areas to both the Sargasso Sea and Mid-Atlantic Ridge is the next step to confirm the success of adults migrating to their spawning area.

The collection of tiny larvae, known as preleptocephali, has been reported for both species in the Sargasso Sea. Preleptocephali are newly hatched larvae less than 6 mm long, and are genetically identified to be American eel, European eel, or other marine eel species. Molecular techniques are indispensable because morphological species identification does not work for undeveloped eggs and preleptocephali. Preleptocephali collected in the Sargasso Sea appear to be approximately one week old after hatching, which seems a too short duration for transportation of eggs and preleptocephali by currents from the newly proposed spawning area to the collection area in the Sargasso Sea. Therefore, it is indeed a fact that eel spawning occurs in the Sargasso Sea. Although eggs and spawning-condition adults have not been collected there, this lack of collection does not mean absence. There has also been no collection of eggs and adults or even preleptocephali outside Sargasso Sea. These apparent “false negatives” may result from insufficient sampling efforts in the Sargasso Sea and Mid-Atlantic Ridge areas as shown by Westerberg et al. 2018^45^. It is also noteworthy that sampling efforts were not necessarily conducted with appropriate timing, place, and sampling methods, for example, with attention to peak spawning season, lunar phase, sampling grid mesh size of sampling grid, etc.

Based on molecular phylogenetic analyses of all anguillid eels, Atlantic eel ancestors were speculated to have invaded the North Atlantic from the Indo-Pacific through the ancient Tethys Sea before the Isthmus of Suez closed 30 million years ago^46^. They established their small migration loop around the coasts of the North Atlantic. They had a spawning area near the Mid-Atlantic Ridge in the narrow ancient North Atlantic that had not yet well expanded, and larvae were transported to Europe and North America randomly. Based on the expansion of the Atlantic Ocean floor, it is likely that the Atlantic eel split into two distinct species, American and European eels, due to the separation of their spawning areas, migration routes, and recruitment places^42^. The segregation of the two spawning areas probably is still the current situation considering the limited hybridization between both species and the introgression from American eels to European eels^47^. Moreover, the introgression force declines from northern to southern Europe, suggesting that spawning may have taken place in the central part of the newly proposed hatching zone near the Mid-Atlantic Ridge. For effective conservation of these endangered species, we must understand Atlantic eel reproductive ecology, including their respective present-day spawning areas and the evolutionary processes of both eel species. The first step in this process is to organize research cruises to enlarge the domain of survey and to validate a newly proposed Mid-Atlantic ridge hypothesis.

## Methods

The HYbrid Coordinate Ocean Model^48^ (HYCOM, https://hycom.org) is a data-assimilative ocean circulation model that provides three-dimensional (3D) currents and hydrological fields used in particle tracking in this study. HYCOM is a primitive equation general circulation model with hybrid vertical coordinates that include isopycnic, terrain-following, and z-coordinates. The global reanalysis v3.0 we used in this work has a 0.08° horizontal resolution and 32 vertical layers. The daily HYCOM reanalysis fields cover October 1992 to December 2012. Surface forcing driving HYCOM includes wind stresses and net heat fluxes and precipitation from the hourly reanalysis produced by the National Centers for Environmental Prediction/Climate Forecast System Reanalysis. Satellite and in situ temperature and salinity data were assimilated into the model based on the Navy Coupled Ocean Data Assimilation System. HYCOM has been validated against observations^49, 50^ and has been widely used to analyze ocean dynamics in the world’s oceans^51, 52^.

We used a 3D particle-tracking method developed by Ohashi and Sheng^53^ to simulate the movement of virtual eel larvae (v-larvae) in this study. The tracking scheme was based on the fourth-order Runge–Kutta method^54^. A random walk displacement was included to represent unresolved sub-grid turbulent flow and other local processes^53^. The estimated maximum horizontal and vertical displacements due to random walk that determined by time step and eddy diffusivity were 600 m and 20 m, respectively. The tracking time step was three hours. The same tracking scheme has been used in previous studies to investigate the migration of Japanese eel larvae and adults in the western Pacific Ocean^55–57^ and has also been applied to simulations of long-distance migration of adult American eels in the Atlantic^58^.

Passive and directional swimming were examined in this study. In the active swimming experiment, two directions (northeast and northwest) were chosen, and the speed was set at 1 BL/s, assuming a linear growth of 0.25 mm/day^8^, and the given swimming speed for 200-day old eel larvae was about 6 cm/s. The orientation abilities of v-larvae were assigned as suggested by recent literature^21, 23^ with American eels and European eel leptocephali to swim northwestward and northeastward respectively. Besides, mortality was not considered in the present study, as the present work attempts to show an example of eel larvae distribution when departing from different areas but not to model the dispersion speeds and timing. Therefore, the simplest settings were used. The North Atlantic Oscillation (NAO) has been suggested to affect Atlantic eel recruitment^59^. The NAO was in a generally positive phase from 1982 to 1995, and turned negative from 1995 to the present. We used an eight-year simulation period (1993–2000) that included positive (1993–1994, 1999–2000) and negative (1995–1996, 1997–1998) NAO events, which could correspond to different ocean conditions, such as shifting of thermal and/or salinity fronts. The results were expressed based on the eight-year composite. We selected a release depth at the upper thermocline (~ 100 m) in reference to where Japanese eel eggs were collected^13^.

The release region was chosen to be the intersection between the Mid-Atlantic Ridge and the two thermal fronts (19–29° N, 47–43° W), or a salinity front (15–19° N, 48–46° W). V-larvae released in historical survey zones in Sargasso Sea were from 23–27° N, 75–62° W (American eel larvae) and 25–29° N, 70–50° W (European eel larvae).

We set the release time during the potential spawning period from February to April^8^, and staggered each release by a 15 day time interval. The estimated migration duration of Atlantic eels was either 17 or 28 months^8^. The tracking duration was set to be 720 days.

The backward tracking scheme was used to find the potential origins of observed eel larvae. In the backward tracking experiment, v-larvae were released where eel larvae have been collected in the Sargasso Sea and tracked passively. Eel larvae of 6–10.9 mm were estimated to be a few days to a few weeks old, thus v-larvae were tracked backward for 30 days. We included random walk in the simulation and we also tested a case without random walk, which had results like the case with random walk.

We calculated the percentages of arrival at 25°W and the Caribbean Sea and the Gulf of Mexico to distinguish recruitment of European and American eels, respectively. The arrival at 25°W counted v-larvae that arrived east of 25°W, whereas arrival to the Caribbean Sea and Gulf of Mexico counted v-larvae that entered those locations.

## References

[1] Schmidt J (1923). Breeding places and migrations of the Eel. Nature.

[2] Tucker DW (1959). A new solution to the Atlantic Eel problem. Nature.

[3] Williams, G. C. & Koehn, R. K. in *Evolutionary Genetics of Fishes* (ed Bruce J. Turner) 529–560 (Springer US, 1984).

[4] Avise, J. C. in *Eel Biology* (eds Katsumi Aida, Katsumi Tsukamoto, & Kohei Yamauchi) 31–48 (Springer Japan, 2003).

[5] Watanabe S, Aoyama J, Tsukamoto K (2004). Reexamination of Ege's (1939) use of taxonomic characters of the genus anguilla. Bull. Mar. Sci..

[6] Wirth T, Bernatchez L (2003). Decline of North Atlantic eels: a fatal synergy?. Proc. Biol. Sci..

[7] Kleckner RC, McCleave JD (1988). The northern limit of spawning by Atlantic eels (Anguilla spp.) in the Sargasso Sea in relation to thermal fronts and surface water masses. J. Mar. Res..

[8] Miller MJ (2015). A century of research on the larval distributions of the Atlantic eels: a re-examination of the data. Biol. Rev..

[9] Boetius, J. & Harding, E. F. A re-examination of Johannes Schmidt's Atlantic eel investigations [*Anguilla rostrata, Anguilla anguilla*, spawning, larvae length (TNV), myomere counts (TNM)]. **v. 4** (1985).

[10] Ayala DJ (2018). Gelatinous plankton is important in the diet of European eel (*Anguilla anguilla*) larvae in the Sargasso Sea. Sci. Rep..

[11] Fricke H, Kaese R (1995). Tracking of artificially matured eels (*Anguilla anguilla*) in the Sargasso Sea and the problem of the eel's spawning site. Naturwissenschaften.

[12] Fricke HDU, Tsukamoto K (1998). Seamounts and the mystery of Eel spawning. Naturwissenschaften.

[13] Tsukamoto K (2011). Oceanic spawning ecology of freshwater eels in the western North Pacific. Nat. Commun..

[14] Chow S (2008). Discovery of mature freshwater eels in the open ocean. Fish. Sci..

[15] Kurogi H (2011). First capture of post-spawning female of the Japanese eel *Anguilla japonica* at the southern West Mariana Ridge. Fish. Sci..

[16] Tsukamoto K (1992). Discovery of the spawning area for Japanese eel. Nature.

[17] Tsukamoto K (2003). Seamounts, new moon and eel spawning: the search for the spawning site of the Japanese eel. Environ. Biol. Fishes.

[18] Ishikawa S (2001). Spawning time and place of the Japanese eel *Anguilla japonica* in the North Equatorial Current of the western North Pacific Ocean. Fish. Sci..

[19] Baltazar-Soares M (2014). Recruitment collapse and population structure of the European eel shaped by local ocean current dynamics. Curr. Biol. CB.

[20] Martin J (2010). An otolith microchemistry study of possible relationships between the origins of leptocephali of European eels in the Sargasso Sea and the continental destinations and relative migration success of glass eels. Ecol. Freshw. Fish.

[21] Naisbett-Jones LC, Putman NF, Stephenson JF, Ladak S, Young KA (2017). A magnetic map leads Juvenile European Eels to the Gulf Stream. Curr. Biol..

[22] Durif CMF (2013). Magnetic compass orientation in the European Eel. PLoS ONE.

[23] Baltazar-Soares M, Eizaguirre C (2017). Animal navigation: the Eel’s magnetic guide to the Gulf stream. Curr. Biol..

[24] Righton D (2016). Empirical observations of the spawning migration of European eels: the long and dangerous road to the Sargasso Sea. Sci. Adv..

[25] Aarestrup K (2009). Oceanic spawning migration of the European Eel (*Anguilla anguilla*). Science.

[26] Beguer-Pon M, Castonguay M, Shan S, Benchetrit J, Dodson JJ (2015). Direct observations of American eels migrating across the continental shelf to the Sargasso Sea. Nat. Commun..

[27] Westin L (1990). Orientation mechanisms in migrating European silver eel (*Anguilla anguilla*): temperature and olfaction. Mar. Biol..

[28] Schabetsberger R (2016). Hydrographic features of anguillid spawning areas: potential signposts for migrating eels. Mar. Ecol. Prog. Ser..

[29] Aida K, Tsukamoto K, Yamauchi K (2003). Eel biology. Springer, Japan.

[30] Kwon Y-O, Riser SC (2004). North Atlantic subtropical mode water: a history of ocean-atmosphere interaction 1961–2000. Geophys. Rese. Lett..

[31] Kwon Y-O, Park J-J, Gary SF, Lozier MS (2015). Year-to-year reoutcropping of eighteen degree water in an eddy-resolving ocean simulation. J. Phys. Oceanogr..

[32] Bultel E (2014). Migration behaviour of silver eels (*Anguilla anguilla*) in a large estuary of Western Europe inferred from acoustic telemetry. Estuar. Coast. Shelf Sci..

[33] Maus S (2009). EMAG2: A 2–arc min resolution Earth Magnetic Anomaly Grid compiled from satellite, airborne, and marine magnetic measurements. Geochem. Geophys. Geosyst..

[34] German CR (2016). Hydrothermal impacts on trace element and isotope ocean biogeochemistry. Philos. Trans. R. Soc. A Math. Phys. Eng. Sci..

[35] Chelton DB, Schlax MG, Samelson RM (2011). Global observations of nonlinear mesoscale eddies. Prog. Oceanogr..

[36] Baltazar-Soares M, Eizaguirre C (2016). Does asymmetric gene flow among matrilines maintain the evolutionary potential of the European eel?. Ecol. Evol..

[37] Als TD (2011). All roads lead to home: panmixia of European eel in the Sargasso Sea. Mol. Ecol..

[38] Pujolar JM (2014). Genome-wide single-generation signatures of local selection in the panmictic European eel. Mol. Ecol..

[39] Dannewitz J (2005). Panmixia in the European eel: a matter of time. Proc. Biol. Sci..

[40] Chang Y-LK, Miller MJ, Tsukamoto K, Miyazawa Y (2018). Effect of larval swimming in the western North Pacific subtropical gyre on the recruitment success of the Japanese eel. PLoS ONE.

[41] Kimura S, Tsukamoto K (2006). The salinity front in the North Equatorial Current: a landmark for the spawning migration of the Japanese eel (*Anguilla japonica*) related to the stock recruitment. Deep Sea Res. Part II.

[42] Tsukamoto K, Aoyama J, Miller MJ (2002). Migration, speciation, and the evolution of diadromy in anguillid eels. Can. J. Fish. Aquat. Sci..

[43] Miller MJ, Tsukamoto K (2017). The ecology of oceanic dispersal and survival of anguillid leptocephali. Can. J. Fish. Aquat. Sci..

[44] Amilhat E (2016). First evidence of European eels exiting the Mediterranean Sea during their spawning migration. Sci. Rep..

[45] Westerberg H (2018). Larval abundance across the European eel spawning area: an analysis of recent and historic data. Fish. Fish..

[46] Aoyama J, Nishida M, Tsukamoto K (2001). Molecular phylogeny and evolution of the freshwater Eel, Genus Anguilla. Mol. Phylogen. Evol..

[47] Wielgoss S, Gilabert A, Meyer A, Wirth T (2014). Introgressive hybridization and latitudinal admixture clines in North Atlantic eels. BMC Evol. Biol..

[48] Chassignet E (2009). US GODAE: global ocean prediction with the HYbrid coordinate ocean model (HYCOM). Oceanography.

[49] Kelly KA, Thompson L, Cheng W, Metzger EJ (2007). Evaluation of HYCOM in the Kuroshio Extension region using new metrics. J. Geophys. Res. Oceans.

[50] Chassignet EP, Smith LT, Halliwell GR, Bleck R (2003). North Atlantic simulations with the hybrid coordinate ocean model (HYCOM): impact of the vertical coordinate choice, reference pressure, and thermobaricity. J. Phys. Oceanogr..

[51] Metzger EJ (2010). Simulated and observed circulation in the Indonesian Seas: 1/12° global HYCOM and the INSTANT observations. Dyn. Atmos. Oceans.

[52] Shu Y (2014). Meridional overturning circulation in the South China Sea envisioned from the high-resolution global reanalysis data GLBa0.08. J. Geophys. Res. Oceans.

[53] Ohashi K, Sheng J (2015). Investigating the effect of oceanographic conditions and swimming behaviours on the movement of particles in the Gulf of St. Lawrence using an individual-based numerical model. Atmos. Ocean.

[54] Press W, Teukolsky S, Vetterling W, Flannery B (1992). Numerical Recipes in Fortran 77: The Art of Scientific Computing.

[55] Chang Y-L, Sheng J, Ohashi K, Béguer-Pon M, Miyazawa Y (2015). Impacts of interannual ocean circulation variability on Japanese Eel Larval Migration in the Western North Pacific ocean. PLoS ONE.

[56] Chang Y-LK, Miyazawa Y, Miller MJ, Tsukamoto K (2018). Potential impact of ocean circulation on the declining Japanese eel catches. Sci. Rep..

[57] Chang Y-L, Miyazawa Y, Béguer-Pon M (2016). Simulating the oceanic migration of silver Japanese Eels. PLoS ONE.

[58] Béguer-Pon M, Ohashi K, Sheng J, Castonguay M, Dodson JJ (2016). Modeling the migration of the American eel in the Gulf of St. Lawrence. Mar. Ecol. Prog. Ser..

[59] Friedland KD, Miller MJ, Knights B (2007). Oceanic changes in the Sargasso Sea and declines in recruitment of the European eel. ICES J. Mar. Sci..

